# Attitudes and practices of health care providers towards improving adherence to smoking cessation medications in Australia: A descriptive study

**DOI:** 10.1002/hpja.674

**Published:** 2022-11-01

**Authors:** Amanual Getnet Mersha, Parivash Eftekhari, Michelle Kennedy, Gillian Sandra Gould

**Affiliations:** ^1^ School of Medicine and Public Health The University of Newcastle Newcastle NSW Australia; ^2^ Hunter Medical Research Institute Newcastle NSW Australia; ^3^ Faculty of Health Southern Cross University Hogbin Drive Coffs Harbour NSW Australia

**Keywords:** adherence, health care provider, quitting, smoking, smoking cessation

## Abstract

**Issue Addressed:**

Adherence to smoking cessation medications is low and predicts the success of quit attempts. Health care providers (HCPs) role in delivering smoking cessation support is crucial. HCPs support to improve adherence to smoking cessation medication has not been evaluated in Australia. This study describes the attitudes and practices of HCPs in Australia towards adherence to smoking cessation medications (nicotine replacement therapies, varenicline and bupropion) and intervention options.

**Methods:**

A descriptive cross‐sectional study was conducted using a convenience sample of 70 HCPs in Australia. Participants were recruited through the social media platforms of professional societies in Australia. Data was collected in the periods between November 2020 and September 2021. Descriptive statistics were performed using SPSS statistical software version 27.0 and data was presented using proportions and percentages.

**Results:**

The majority of participants were doctors, nurses and midwives (82.8%). Almost two‐thirds of the participants (68.6%) self‐reported that they provided adequate adherence support to individuals taking smoking cessation medications. The majority of participants (87.1%) identified adherence support service as part of their professional role. Only 11.1% of the participants who did not believe supporting medication adherence to be their role reported providing adherence support. The main perceived barriers to adherence support are lack of skill, knowledge, time and resources. HCPs believed that providing additional counselling and monitoring of adherence can improve adherence rates.

**Conclusions:**

In an online survey conducted in Australia, HCPs indicated multiple barriers to providing adherence support and intervention strategies that should be considered for smoking cessation programs. A higher proportion of participants who perceived adherence support as their professional role reported supporting adherence to smoking cessation medications.

**So What?:**

Considerations should be given to improve HCPs attitudes and practices towards smoking cessation medications adherence support. Smoking cessation programs should consider the issue of adherence support. Further studies with a larger sample size across a broader range of HCPs are needed to extensively understand adherence service provision among HCPs in Australia.

**Abbreviation**
HCPs: health care providers

## 1 BACKGROUND

Effective smoking cessation medications are available to support quitting.^1^, ^2^ However, the use of smoking cessation medications such as nicotine replacement therapies (NRT), varenicline and bupropion was found to be inconsistent based on the type of medication, study design, adherence assessment time point and participant characteristics.^3^ Adherence to NRT ranges from as low as 16% in a 2005 cross‐sectional study conducted among predominantly male (80%) Chinese smokers who attended an outpatient clinic^4^ to 68% in a 2007/2008 study conducted in Syria among participants of a randomised controlled trial.^5^ Previous studies reported that half of the smokers trying to quit smoking and enrolled in randomised controlled trials who were provided with varenicline,^6^, ^7^ or bupropion^8^ were nonadherent to their treatment. The level of adherence to smoking cessation medication (NRT, varenicline and bupropion) among smokers and ex‐smokers in Australia was reported to be 28.4%^9^ and adherence increased successful quitting by 2‐fold.^10^

Multiple psychosocial, environmental and medication‐related factors affect adherence to smoking cessation medications.^11^ Barriers affecting adherence to smoking cessation medications can be classified as patient‐related and health care provider‐related factors.^11^, ^12^ Patient‐related factors include forgetfulness, misinformation about smoking cessation medications, alcohol intake, withdrawal symptoms, exposure to triggers and others. Additionally, adherence to smoking cessation medications can be affected by the adequacy of the service received from health care providers (HCPs).^13^, ^14^ A recent finding from a national cross‐sectional survey in Australia demonstrated that experiencing psychological disorders, and medication‐related factors such as the use of smoking cessation medications (NRT, varenicline and bupropion) in previous quit attempts were the main predictors of adherence.^9^

Previous studies conducted among HCPs focused on a more general smoking cessation service rather than detailing the components of service provision such as medication adherence support.^13^, ^14^ Studies conducted in Australia indicated that the majority of HCPs assessed smoking characteristics, but rates of providing cessation support were reported to be low.^15^, ^16^ The most common factors reported to affect smoking cessation provision were lack of knowledge, skill and resources.^15^, ^17^ In 2017, a national cross‐sectional study was conducted in the Netherlands to evaluate the practice of smoking cessation service provision among 883 HCPs including addiction specialists, general practitioners, internists, neurologists, pulmonologists, midwives and others.^18^ The largest proportion of participants were general practitioners (16.7%) and pulmonologists (11.5%). The most common reasons given by HCPs for not providing adequate support include lack of time, lack of training and perceived lack of motivation in patients. Clinicians who perceived that smoking cessation service was a core component of their role were more likely to implement most components of smoking cessation care.^18^ In 2014, a qualitative study explored the perceptions of HCPs about smoking cessation support. The study illustrated that treating physicians and nurses did not provide detailed smoking cessation support including medication‐specific issues such as adherence and addressing side effects. This could be due to time constraints and a lack of comprehensive knowledge.^19^ Furthermore, studies showed that many smokers have never been advised about smoking and smoking cessation support during their visit to health facilities for other reasons.^19^

HCP‐delivered interventions directed to improve medication adherence were found to improve the rate of adherence to smoking cessation medications and the success of quit attempts.^20^, ^21^, ^22^, ^23^, ^24^ Studies variously provided: additional counselling focusing on the issue of medication adherence^24^; motivational interviewing techniques^20^, ^21^; electronic medication monitoring,^23^ and specific feedback that may improve adherence to smoking cessation medications. Moreover, interventions directed to address participants' concerns about the safety and efficacy of smoking cessation medications and sending daily reminders improved adherence to smoking cessation medications.^20^, ^25^ A Cochrane review including the above‐mentioned trials was conducted in 2019 to evaluate the effectiveness of interventions directed to improve adherence to smoking cessation medications such as nicotine patches, nicotine gum and bupropion. The review found a modest improvement in the rate of adherence to smoking cessation medications. Self‐reported and biochemically validated rates of short‐term (less than 6 months) and long‐term smoking abstinence (6‐12 months) were found to be higher in the intervention groups.^26^

HCPs play an essential role in facilitating smoking cessation^27^ and they are at the core of medication adherence interventions and implementation studies. Although there are studies that evaluated the attitudes and practices of HCPs in delivering smoking cessation care in Australia,^15^, ^16^, ^17^ there is a lack of evidence about providing support for adherence to smoking cessation medications. To the best of our knowledge, there has been no study conducted in Australia aimed to evaluate the provision of HCP support for adherence to smoking cessation medications (NRT, varenicline and bupropion). Hence, exploring HCPs‐related factors will provide essential input into designing effective interventions that can improve adherence to smoking cessation medications and improve quit rates.

## 2 METHODS

A descriptive cross‐sectional study was conducted among Australian HCPs (such as physicians, nurses, midwives, pharmacists, psychologists, addiction specialists and tobacco treatment specialists) to assess their attitudes, perceived barriers and practices regarding adherence to smoking cessation medications. This study was approved by the University of Newcastle Human Ethics Committee, ethics approval number H‐2020‐0334, and informed consent was taken from the participants.

### 2.1 Participant recruitment and data collection

Data collection was conducted in the periods between November 2020 and September 2021. A convenience sample of 70 health care professionals (physician, nurse, midwife, pharmacist, psychologist, addiction specialist and Aboriginal health worker) currently working in Australia were included in the study. Data was collected in an anonymous manner and participants were informed that the obtained information will be kept anonymous and recorded in such a way that the respondent could never be identified. Recruitment of study participants was carried out using online social media platforms of professional networks in Australia such as the Royal Australian College of General Practitioners (RACGP), Australasian Professional Society on Alcohol and other Drugs (APSAD), Australian Chapter of Addiction Medicine (AChAM) and other relevant societies.

### 2.2 Survey instrument

This study used existing literature and input from experts in the area of smoking cessation to develop the survey tool to assess the attitudes and practices of HCPs regarding adherence to smoking cessation medications.^13^, ^14^, ^18^ The survey instrument is included in Supplementary Material 1.

The first section assesses HCPs demographic characteristics such as gender (male or female), profession (general practitioner, gynaecologist and obstetrician, internist, surgeon, paediatrician, nurse, midwife, pharmacist, clinical psychologist, addiction specialist, aboriginal health worker, tobacco treatment specialist), years of professional work experience and age of participants. The second section evaluated attitudes about adherence and adherence support provision using Likert scales. We assessed the frequency of adherence support during follow‐up visits (always, frequently, occasionally, rarely, never), providers' belief about their role in adherence support (strongly agree, agree, neutral, disagree, strongly disagree), perceived patient adherence rates (0%‐25%, 26%‐50%, 51%‐75%, 76%‐100%) and perceived patient honesty regarding medication adherence (always, frequently, occasionally, rarely, never).

The third section assessed perceived barriers to adequate adherence support provision using a five‐point Likert scale^1^: strongly disagree^2^; disagree^3^; neither agree nor disagree^4^; agree^5^; strongly agree. The assessed factors were lack of skill, lack of knowledge, lack of time, lack of the necessary resources and the perceived role of adherence support. The final (4th) section assessed the attitude of HCPs about different strategies targeted at improving adherence to smoking cessation medications by using a five‐point Likert scale^1^: strongly disagree^2^; disagree^3^; neither agree nor disagree^4^; agree^5^; strongly agree. The following interventions were evaluated: additional counselling about smoking cessation medications and adherence; motivational interviewing; feedback and offering financial incentives. Moreover, we assessed what HCPs believed might provide a more appropriate way to deliver adherence support: face‐to‐face interventions; web‐based interventions; short text messages; mobile applications; mobile phone call‐based interventions.

### 2.3 Data analysis

Data was entered into SPSS statistical software version 27.0 for further data management and analysis. Descriptive statistics were calculated to characterise HCPs characteristics, adherence support, perceived barriers to adherence support provision and potential interventions to improve adherence. Findings were presented using proportions, percentages, means and standard deviations. Adherence support practice was dichotomised into adequate and nonadequate support.

Variables were presented after dichotomisation to conduct a cross‐tabulation and present percentages based on participant characteristics. Adherence support practice was dichotomised into adequate and nonadequate support. Adequate adherence support practice is defined in this study if participants provided medication adherence support frequently or always during encountering individuals using smoking cessation medications for the purpose of quitting. Likert scales were recategorised as “agree” if participants' choice “strongly agrees” or “agree” for the item and “disagree” if “strongly disagree,” “neutral,” or “disagree.”

Cross‐tabulation and chi‐square statistical tests were conducted to evaluate the association between adherence support practice and various demographic and attitudes towards adherence to smoking cessation medications. To conduct a chi‐square statistical test the following factors have been recategorised as follow: profession (Doctors or other HCPs including nurses and midwives); age (≤35 years old or >35 years old); professional work experience (≤10 years or >10 years); perceived adherence rate (HCPs who believe >50% or ≤50% are adherent to smoking cessation medications). Measures of association between categorical variables were assessed using the chi‐square test, with a *P*‐value <.05 indicating a statistically significant association. Due to the low number of participants, a further statistical analysis such as logistic regression was not conducted.

## 3 RESULTS

### 3.1 Participant characteristics

A total of 70 HCPs completed the questionnaire. Demographic information is illustrated in Table 1. The participants consisted of a range of HPs including doctors (35.7%), nurses and midwives (47.1%), pharmacists (5.7%) and other HPs such as clinical psychologists and tobacco treatment specialists (11.4%). The median age of participants is 40 years with a mean age of 42.5 ± 13.4 years. The median and mean years of professional experience for the participants are 16 and 18.1 ± 13.4 years, respectively. The majority (59.3%) of male participants were doctors as compared to 20.9% for female participants. A quarter of male participants (25.9%) and the majority 60.5% of female participants were nurses and midwives (Table 1).

**TABLE 1:** Characteristics of participating health care providers (N = 70)

Variables	Frequency (%)
Gender	
Male	27 (38.6%)
Female	43 (61.4%)
Age (mean ± SD)	42.5 ± 13.4 years
Years in practice (mean ± SD)	18.1 ± 13.4 years
Profession	
Doctors	25 (35.7%)
Nurses and midwives	33 (47.1%)
Pharmacists	4 (5.7%)
Other HPs	8 (11.4%)
Frequency of adherence support	
Always	20 (28.6%)
Frequently	28 (40%)
Occasionally	10 (14.3%)
Rarely	7 (10%)
Never	5 (7.1%)
Perceived adherence rate	
0%‐25%	28 (40%)
26%‐50%	32 (45.7%)
51%‐75%	9 (12.9%)
76%‐100%	1 (1.4%)
Perceived patient honesty about medication use	
Always	9 (12.9%)
Frequently	34 (48.6%)
Occasionally	21 (30%)
Rarely	6 (8.6%)
Believe it is my role to provide adherence support	
Strongly agree	32 (45.7%)
Agree	29 (41.4%)
Neutral	5 (7.1%)
Disagree	3 (4.3%)
Strongly disagree	1 (1.4%)

### 3.2 Attitude and practice of HCPs regarding medication adherence service provision

Two‐thirds of the participants (68.6%) reported that they provided adherence support frequently or always during supporting individuals taking medicines to support their quitting attempt. The majority of the participants (85.7%) believed less than 50% of individuals they supported were nonadherent to smoking cessation medications. Moreover, 61.4% of participants believed that smokers were honest about reporting about their medication adherence. More male participants (77.8%) than female participants (62.8%) reported providing adequate adherence support.

The majority of participants (87.1%) believed that adherence support provision is their role. Whereas 12.9% of participants do not believe it is their role to provide adherence support. All of the doctors and pharmacists believed that the provision of adherence support was their role. Among the nurses and midwives, 78.8% believed the provision of adherence support was their role. In regard to the health profession of participants and adherence support, the majority of the doctors (80%), 69.7% of the nurses and midwives and 25% of pharmacists reported that they always or frequently asked and supported medication adherence during their clients' visit for a quit attempt.

Among the participating HCPs who believed adherence support is part of their role (87.1%), 77% self‐reported that they have provided adherence support frequently or always. Whereas 11.1% of the HCP who did not believe adherence support as part of their role (13.4%) reported supporting adherence always or frequently. Among the participants, 90% of HCPs who perceived more than 50% of smokers are adherent to their medications, provided adherence support. While 65% of participants who perceived less than 50% of smokers are adherent to their medicines provide adherence support.

Chi‐square tests were performed to see the association between adherence support provision practice and various variables (gender, age, professional work experience, role belief, perceived adherence rate). Categories for participants' age and role‐belief about adherence support provision were found to be significantly associated with adherence support provision (chi‐square [χ^2^] statistic *P*‐value <.05) (Table 2).

**TABLE 2:** Association between adherence support provision with participant demographic and attitude towards adherence to smoking cessation medications (N = 70)

Variable	Adherence support		Chi *P*‐value
	Adequate	Nonadequate	
Gender			.189
Male	21 (77.8%)	6 (22.2%)	
Female	27 (62.8%)	16 (37.2%)	
Age			.012*
≤35 y old	10 (47.6%)	11 (52.4%)	
>35 y old	36 (78.3%)	10 (21.7%)	
Professional work experience			.087
≤10 y	15 (57.7%)	11 (42.3%)	
>10 y	31 (77.5%)	9 (22.5%)	
Profession			.082
Doctors	20 (80%)	5 (20%)	
Nurses and midwives	23 (69.7%)	10 (30.3%)	
Pharmacists	1 (25%)	3 (75%)	
Others	4 (50%)	4 (50%)	
Perceived adherence rate			.086
≤50%	39 (65%)	21 (35%)	
>50%	9 (90%)	1 (10%)	
Perceived patient honesty about medication use			.063
Always/frequently	33 (76.7%)	10 (23.3%)	
Sometimes/never/occasionally	15 (55.6%)	12 (44.4%	
Believe it is my role to provide adherence support			.001*
Agree	47 (77%)	14 (23%)	
Disagree	1 (11.1%)	8 (88.9%)	

* *P*‐values obtained from the chi‐square test is less than .05.

### 3.3 Perceived barriers of adherence support provision

**FIGURE 1:**
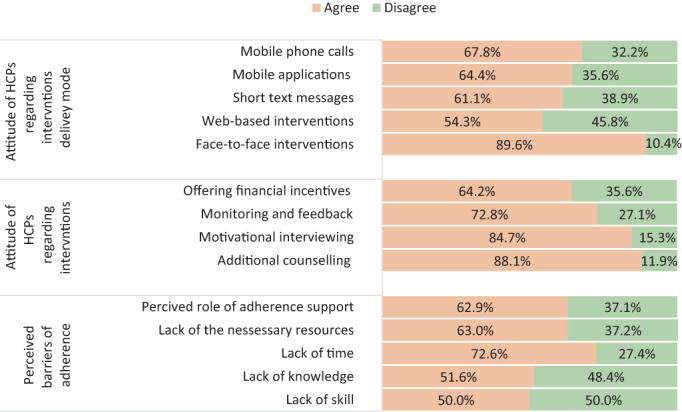
Perceived barriers and interventions to improve adherence to smoking cessation medications. “Agree”—participants who believed the specific intervention strategies and intervention deliver options can result in improved adherence to smoking cessation medications. For the perceived barriers, “Agree” stands for the percentage of participants who perceived the specific factor is a barrier to adherence support provision. HCPs, health care providers

Half of the participants reported they “strongly agree” or “agree” that lack of skill (50%) and knowledge (51.6%) had affected the range of adherence support they provide. Lack of time (72.6%) and resources (63%) was stated as a reason for inadequate support towards adherence to smoking cessation medications. Among the survey participants, 62.9% reported they “strongly agree” or “agree” that role belief affects adherence support provision (Figure 1).

### 3.4 Attitude of HCPs regarding interventions targeted at improving adherence

Participants were asked if they believe that the presented intervention strategies and modes of intervention delivery options are effective in improving adherence to smoking cessation medications. “Agree” is defined if the participant chooses “strongly agree” or “agree” for the presented strategies or modes of intervention delivery options.

Strategies agreed to by the participants for improving adherence were: additional medication adherence counselling (88.1%), motivational interviewing (84.7%), monitoring and feedback (72.8%) and financial incentives (64.2%) (Figure 1).

Modes of delivery for medication adherence‐related interventions agreed to by the participants were: face‐to‐face intervention (89.6%), phone calls (67.8%), mobile applications (64.4%), short text messages (61.1%) and web‐based intervention (54.3%) (Figure 1).

## 4 DISCUSSION

The current study examined the attitudes, perceived barriers and practices of HCPs towards providing support for adherence to smoking cessation medications in Australia. The study also evaluated the attitudes of HCPs regarding various intervention strategies directed to improve adherence to smoking cessation medications. A range of health professionals such as doctors, nurses, midwives and pharmacists were included in this study.

Developing a positive attitude towards adherence to smoking cessation medication and providing support is essential to improve treatment effectiveness and outcomes. Having a positive attitude that medication adherence can be improved and it affects clinical outcomes was reported to improve the support that HCPs provided.^28^ Two‐thirds of the HCPs participated in this study reported they have provided adequate adherence support during contacting individuals taking medicines to ease quitting. Adherence support is higher among participants who believed that adherence support is part of their role. In light of this, training for HCPs to improve their attitude towards adherence to medicines incorporated with available smoking cessation training is recommended. More male participants self‐reported providing adequate adherence support compared to female participants. This could be due to the difference in gender of participants based on profession that is, more male participants in the sample were doctors and all of the participating doctors believe adherence support is part of their professional role, which may have contributed to the difference in service provision. A study conducted among HCPs in the Netherlands showed role belief as one of the essential components for smoking service care.^18^ In this study, the majority of the participants perceived that the rate of adherence to smoking cessation medications is less than 50%. This finding is consistent with a recent study conducted in Australia that showed adherence rate of 28.4%.^9^

Participants mentioned multiple perceived barriers to adherence support provision. The most commonly reported barriers were lack of time and resources. Previous studies conducted to evaluate factors associated with smoking cessation service provision reported similar findings regarding the lack of necessary resources and time constraints. Thus, health care facilities are recommended to make the resources needed by HCPs available such as reading materials and commonly used medicines such as nicotine patches and gum.^18^ Lack of essential knowledge and skill were also reported by half of the participants. Lack of training and time were similarly reported by other studies as the main factors associated with the effective implementation of smoking cessation care.^18^, ^29^, ^30^, ^31^ Lack of time can be both a real or perceived barrier that can be improved with training and creating a positive role belief. Considering that participation in training is also affected by interest and time constraints, it is essential to prepare smoking cessation training in a more context‐specific and relevant way.^18^ Often it takes multiple attempts before successfully quit smoking.^32^ Providing adequate adherence support could improve the rate of smoking cessation and help reduce the number of quit attempts before being successful and saves time for HCPs.^26^ Thus, HCPs trainings are recommended to integrate information about the time they can save because of clients who achieved quitting with fewer number of quit attempts. Furthermore, providing smoking cessation care at different levels of health care including primary care settings, and increasing the workforce can improve the provision of appropriate adherence support.^33^

Providing training, guidelines and online resources targeted at adherence service provision are essential to improve smoking cessation care.^34^ Role belief was reported by more than half of the participants as a barrier to adherence service provision. Participants with stronger role identities from other studies were found to use appropriate smoking cessation guidelines and were motivated to provide adequate support.^18^ In‐depth discussions and incentivisation are also recommended to create a positive attitude towards adherence support.^35^, ^36^

Participants agreed to several intervention options and modes of intervention administration strategies to potentially improve adherence to smoking cessation medications. The vast majority of participants believed that providing additional adherence counselling and motivating individuals during their quit smoking attempt can improve adherence rates. Medication use monitoring and providing specific feedback were the main intervention strategies agreed to improve the adherence rate. Face‐to‐face and mobile phone calls, mobile applications and short text message‐based interventions were considered acceptable by more than half of the participants. A Cochrane review evaluated interventions to improve adherence to smoking cessation medications such as electronic medication monitoring and providing specific feedback,^23^ focusing on additional counselling^24^ and sending reminder short text messages.^20^, ^25^ The review indicated a slight improvement in medication adherence and long‐term smoking cessation rates in the intervention groups with odds of 1.16.^26^ An umbrella review of systematic reviews indicated higher rates of smoking cessation among participants whose quit attempts were supported by multicomponent modalities such as a combination of face‐to‐face counselling, short text messages and internet‐based support.^37^

### 4.1 Limitations of the study

Although this study is the first study to evaluate the attitudes and practices of HCPs towards providing services for adherence to smoking cessation medications in Australia, there are several limitations that should be considered during the interpretation of the findings. Data was collected during the coronavirus (COVID‐19) pandemic crisis and was impacted due to a reduction in onsite activities such as face‐to‐face data collection.^38^ One of the limitations in conducting an online survey using professional societies' social media platforms is the difficulty in determining the exact denominator to calculate the response rate.^39^, ^40^ The sample size was low and may not represent the actual situation of service provision practices. Adherence support practice was measured via self‐report and thus may be subject to reporting bias. As this is a descriptive study, we were not able to explore the associations between different factors and the practice of medication adherence support provision. Considering the reportedly high rates of burnout among HCP due to the impact of the COVID‐19 pandemic on the health system and improve survey completion rates,^41^ emphasis was given to exploring the attitude and barriers to smoking cessation medication adherence support. This study would benefit from replication using a more representative sample with a comprehensive tool to explore both barriers and facilitators to adherence support provision.

## 5 CONCLUSIONS AND RECOMMENDATIONS

This study showed that the majority of HCPs surveyed self‐reported that they provided support to improve adherence to smoking cessation medication. Time constraints, lack of knowledge, lack of skills, limitation of resources and role‐belief were the main perceived barriers to adherence support provision. Although formal hypothesis testing was not conducted in this study, the majority of participants who perceived adherence support as their professional role reported supporting adherence to smoking cessation medications. HCPs agreed to multiple intervention strategies that should be considered in smoking cessation programs to raise adherence. Training is recommended to enhance the knowledge and skills of HCPs in providing effective medication adherence support. Finally, more research is needed to fully understand the issues relevant to smoking cessation medication and adherence service provision among HCPs using larger and more representative data.

## AUTHOR CONTRIBUTIONS

*Conceptualisation*: Amanual Getnet Mersha. *Methodology*: Amanual Getnet Mersha, Parivash Eftekhari, Michelle Kennedy and Gillian Sandra Gould. *Formal analysis*: Amanual Getnet Mersha. *Investigation*: Amanual Getnet Mersha, Parivash Eftekhari, Michelle Kennedy and Gillian Sandra Gould. *Writing—original draft preparation*: Amanual Getnet Mersha. *Writing—revised version and editing*: Amanual Getnet Mersha, Parivash Eftekhari, Michelle Kennedy and Gillian Sandra Gould. *Supervision*: Parivash Eftekhari, Michelle Kennedy and Gillian Sandra Gould. All authors have read and agreed to the published version of the manuscript.

## CONFLICT OF INTEREST

The authors declare no conflict of interest.

## Supporting information


**Appendix S1** Supporting Information.

## Data Availability

All relevant materials and data supporting the findings of this study are contained within the manuscript. However, if you need additional information, you can access the data from the corresponding author on a responsible request.
