# Supplementary material for: Attitudes and practices of health care providers towards improving adherence to smoking cessation medications in Australia: A descriptive study
Source: Health Promot J Austr. 2022 Nov 1;34(4):848–55. doi: 10.1002/hpja.674 (PMC10947351; doi:10.1002/hpja.674)
Supplement: Supplementary file 1 — Appendix S1 Supporting Information. [file HPJA-34-848-s001.docx]

# Questionnaire

**Part 1: Demographic characteristics and health care providers’ practice and perspectives about smoking cessation medications**

| **Questions** | **Possible answers** |
| --- | --- |
| 1. Gender | Male  Female |
| 1. Age | …………………Years old |
| 1. Years of since health professional qualification | …………………Years |
| 1. Smoking cessation service Provider type | General practitioner  Gynaecologist and Obstetrician  Internist  Surgeon  Paediatrician  Nurse  Midwife  Pharmacist  Psychologist  Addiction specialist  Aboriginal health worker  Others  If others, please state here………………… |
| 1. Population you are serving | Indigenous Australian  Non-indigenous Australians and other citizens  Both  If both, percentage estimate of indigenous and non-indigenous people ………………… |
| 1. How frequently do you ask about adherence to smoking cessation medications? | Always  Frequently  Occasionally  Rarely  Never |
| 1. What percentage of your patients do you think are adherent to smoking cessation medications? | 0 – 25%  26 – 50%  51 – 75%  76 – 100% |
| 1. How frequent do you think your patients are being truthful about their adherence to smoking cessation medications? | Always  Frequently  Occasionally  Rarely  Never |
| 1. Do you believe it is your role to discuss adherence to smoking cessation medications? | Strongly agree  Agree  Neutral  Disagree  Strongly disagree |

**Part 2: To what extent do you believe the following health care provider related factors affect provision of smoking cessation medication adherence support?**

|  | **Strongly agree** | **Agree** | **Neutral** | **Disagree** | **Strongly disagree** |
| --- | --- | --- | --- | --- | --- |
| Lack of time |  |  |  |  |  |
| Lack of necessary materials such as medications, reading materials |  |  |  |  |  |
| Patient dishonesty about adherence |  |  |  |  |  |
| Believing it is my role to provide medication adherence counselling and monitoring |  |  |  |  |  |
| Lack of adequate skill |  |  |  |  |  |
| Lack of necessary knowledge |  |  |  |  |  |
| If others, please write |  | | | | |

**Part 3: Do you think these interventions will improve adherence to smoking cessation medication?**

|  | **Strongly agree** | **Agree** | **Neutral** | **Disagree** | **Strongly disagree** |
| --- | --- | --- | --- | --- | --- |
| Providing additional counselling about smoking cessation medications |  |  |  |  |  |
| Motivational interviewing |  |  |  |  |  |
| Financial incentives |  |  |  |  |  |
| Automated medication adherence Calls |  |  |  |  |  |
| Providing feedback |  |  |  |  |  |
| If others, please write |  | | | | |

**Part 4:** **To what extent do you believe the following intervention delivery options will be effective in improving adherence to smoking cessation medications?**

|  | **Strongly agree** | **Agree** | **Neutral** | **Disagree** | **Strongly disagree** |
| --- | --- | --- | --- | --- | --- |
| Face-to-face additional medication adherence Counselling |  |  |  |  |  |
| Internet-based interactive supports using such as online chat rooms, Facebook groups, twitter, e mails |  |  |  |  |  |
| Daily text message tips and reminders |  |  |  |  |  |
| Smartphone applications providing tips and reminders |  |  |  |  |  |
| Interactive phone calls focused on smoking cessation medication adherence |  |  |  |  |  |
| If others, please write |  | | | | |
